# A methodological survey of the analysis, reporting and interpretation of Absolute Risk ReductiOn in systematic revieWs (ARROW): a study protocol

**DOI:** 10.1186/2046-4053-2-113

**Published:** 2013-12-13

**Authors:** Pablo Alonso-Coello, Alonso Carrasco-Labra, Romina Brignardello-Petersen, Ignacio Neumann, Elie A Akl, Xin Sun, Bradley C Johnston, Matthias Briel, Jason W Busse, Demián Glujovsky, Carlos E Granados, Alfonso Iorio, Affan Irfan, Laura M García, Reem A Mustafa, Anggie Ramirez-Morera, Iván Solà, Kari A O Tikkinen, Shanil Ebrahim, Per O Vandvik, Yuqing Zhang, Anna Selva, Andrea J Sanabria, Oscar E Zazueta, Robin W M Vernooij, Holger J Schünemann, Gordon H Guyatt

**Affiliations:** 1 Iberoamerican Cochrane Centre, Institute of Biomedical Research (IIB-Sant Pau), Barcelona, Spain; 2 Department of Clinical Epidemiology & Biostatistics, McMaster University, Hamilton, Canada; 3 Evidence-Based Dentistry Unit, Faculty of Dentistry, Universidad de Chile, Santiago, Chile; 4 Department of Medicine, Pontificia Universidad Católica de Chile, Santiago, Chile; 5 Department of Internal Medicine, American University of Beirut, Beirut, Lebanon; 6 Department of Medicine, State University of New York, Buffalo, NY, USA; 7 Chinese Evidence-based Medicine Centre, West China Hospital, Sichuan University, Chengdu, China; 8 Department of Anaesthesia and Pain Medicine, The Hospital for Sick Children, Institute of Health Policy, Management and Evaluation, University of Toronto, Toronto, Ontario, Canada; 9 Child Health Evaluative Sciences, The Hospital for Sick Children Research Institute, Toronto, Ontario, Canada; 10 Basel Institute for Clinical Epidemiology and Biostatistics, University Hospital Basel, Basel, Switzerland; 11 Department of Anaesthesia, McMaster University, Hamilton, Canada; 12 Argentine Cochrane Centre IECS (Institute for Clinical Effectiveness and Health Policy), Buenos Aires, Argentina; 13 Área de investigaciones, Facultad de Medicina, Universidad de La Sabana, Chía, Colombia; 14 Internal Medicine Residency Program, University of Illinois, Urbana-Champaign, Illinois, USA; 15 Department of Medicine and Nephrology, University Of Missouri-Kansas City, Missouri-Kansas City, Missouri, USA; 16 CCSS Permanent Medical Advisor, Health Care Development Division, IHCAI Foundation & Central America Cochrane, Costa Rica, Costa Rica; 17 Department of Urology, Helsinki University Central Hospital and University of Helsinki, Helsinki, Finland; 18 Institute of Health Policy, Management & Evaluation, University of Toronto, Toronto, Canada; 19 Norwegian Knowledge Centre for the Health Services, Oslo, Norway

**Keywords:** Systematic reviews, Meta-analysis, Statistical data, Evidence-based medicine, Numbers needed to treat, Data reporting, Absolute effect measures

## Abstract

**Background:**

Clinicians, providers and guideline panels use absolute effects to weigh the advantages and downsides of treatment alternatives. Relative measures have the potential to mislead readers. However, little is known about the reporting of absolute measures in systematic reviews. The objectives of our study are to determine the proportion of systematic reviews that report absolute measures of effect for the most important outcomes, and ascertain how they are analyzed, reported and interpreted.

**Methods/design:**

We will conduct a methodological survey of systematic reviews published in 2010. We will conduct a 1:1 stratified random sampling of Cochrane vs. non-Cochrane systematic reviews. We will calculate the proportion of systematic reviews reporting at least one absolute estimate of effect for the most patient-important outcome for the comparison of interest. We will conduct multivariable logistic regression analyses with the reporting of an absolute estimate of effect as the dependent variable and pre-specified study characteristics as the independent variables. For systematic reviews reporting an absolute estimate of effect, we will document the methods used for the analysis, reporting and interpretation of the absolute estimate.

**Discussion:**

Our methodological survey will inform current practices regarding reporting of absolute estimates in systematic reviews. Our findings may influence recommendations on reporting, conduct and interpretation of absolute estimates. Our results are likely to be of interest to systematic review authors, funding agencies, clinicians, guideline developers and journal editors.

## Background

When contemplating a recommendation, either in favour of or against an intervention, guideline developers need to consider the balance between desirable and undesirable consequences of treatment alternatives [1]. Also, clinicians and patients seeking shared decision-making need to understand the magnitude of benefits and harms.

Measures of effect of an intervention on dichotomous outcomes may express a change in risk in relative terms (that is, risk ratio, also known as relative risk and relative risk reduction (RRR)), in absolute terms (that is, absolute risk reduction (ARR), also known as risk difference), or as the inverse of the risk difference (that is, the number needed to treat (NNT)) [2].

The impact of an intervention may appear small or large depending on which measure of effect is reported, an issue that is referred to as statistical framing [2,3]. Clinicians are more inclined to prescribe a drug that reduces the relative risk of death by 50% than one that reduces the absolute risk of death from 2% to 1%, or that requires 100 patients to be treated to prevent a single premature death [4,5]. This finding is in spite of the fact that these three presentations (RRR, ARR and NNT, respectively) express the same effect. Similarly, patients are more willing to start a lipid-lowering drug when benefit is presented as a RRR versus an ARR [6].

Empirical evidence suggests that relative effect measures are, on average, more consistent than absolute measures [7,8]. Indeed, studies in patients with differing severities of disease, or studies with different lengths of follow-up, will almost certainly have varying risk differences due to varying baseline risks. For this reason, it is wise to avoid performing meta-analyses directly on risk differences, unless there is a clear reason to suspect that risk differences will be consistent in a particular clinical situation [9,10].

To report one or more absolute effects in accompaniment to relative effects, systematic review authors should apply the measure of relative effect to a baseline risk or control group risk. This involves expressing the absolute difference for each clinically identifiable risk group, and clarifying the time period to which this applies. Consequently, a relative risk is expressed as a variety of risk differences or NNTs across a range of control risks in subpopulations that clinicians can easily identify [8,9,11]. GRADE [11,12], which represents an emerging consensus for rating the quality of the evidence, suggests that when summarizing the evidence, together with the best estimate of relative effects, authors present the best estimates of absolute risks in intervention and control groups and the difference in the two risks, with the corresponding confidence intervals that convey the precision of estimates. Estimates of absolute risk should be provided in this manner consistently for both benefits and harms or burdens. The Cochrane Collaboration provides similar guidance [13].

An analysis of the top general medical journals showed that 68% of randomized controlled trials (RCTs) and cohorts failed to report absolute risks in the abstract. Of these articles, about half did report the underlying absolute risks elsewhere in the article (text, table or figure) but half did not report them anywhere [14]. More recently, a study found that research articles published in top journals in the field of health inequalities reported both relative and absolute effects only 7% of the time in the full text and 2% in the abstract [15].

In the case of systematic reviews in three leading medical journals (*Lancet*, *JAMA* and *BMJ*) Sedrakyan and Shih [16] reported that authors fail to include absolute estimates. Additionally, in a research letter, Beller *et al.*[17] reported that only 4% of systematic reviews include both absolute and relative estimates of effect in the abstract. These analyses have come from a relatively limited sample of journals and only explored the reporting of estimates. Given the lack of information about this topic and the potential implications for decision-making in healthcare [18], it is important to explore how this issue is managed in published systematic reviews.

The objectives of this study are to evaluate the proportion of systematic reviews that report absolute measures of effect for the most important outcome, and ascertain how are they calculated, reported and interpreted. Additionally, the study will evaluate the frequency of mismatched framing, that is, the use of relative measures for benefit outcomes and absolute measures for harm outcomes.

## Methods/design

### Design overview

We will conduct a methodological survey of Cochrane and non-Cochrane systematic reviews. We will use standard methodology for conducting systematic reviews [13], as described in previous protocols from our group [19-22]. We did not register the project in the PROSPERO database.

### Definitions

The risk of an outcome in a group is the proportion of individuals in that group who suffer that outcome. Measures of effect (whether relative or absolute) express the risk of an outcome in one group compared with another. As relative measures of effect, the relative risk is the ratio of the risk of an outcome, whereas the odds ratio (OR) is the ratio of the odds of an outcome [8]. As an absolute measure of effect, the risk difference is the difference between the observed risk in the experimental and control groups. It can also be expressed as the arithmetic difference between two outcome rates. The NNT, another absolute measure of effect, is the inverse of the risk difference, which translates into the number of subjects who need to be treated to prevent one additional outcome, good (NNT) or bad (number needed to harm).

Cochrane systematic reviews are defined as all systematic reviews published in the *Cochrane Database of Systematic Reviews*. All the other systematic reviews will be considered non-Cochrane systematic reviews.

### Eligibility criteria

We will include systematic reviews published in English meeting the following criteria:

1. Described as a ‘systematic review’ or a ‘meta-analysis’;

2. Reports a search strategy in at least one database;

3. Published in 2010; in the Cochrane Database of Systematic Reviews or indexed in MEDLINE;

4. Includes a comparison of an intervention with another intervention or no intervention in human beings;

5. Reports measures of effect for at least one dichotomous outcome either from a single study or from a pooled analysis.

If there is more than one pairwise comparison, reviewers will select the comparison that reports the largest number of dichotomous outcomes. If more than one comparison reported the same number of dichotomous outcomes, reviewers will select the comparison that reports the largest number of absolute estimates. We will identify the most patient-important outcome using a hierarchical approach (Appendix 1). If the outcome is a composite outcome, we will select the most patient-important of those included in the composite, if authors provide disaggregated data in the review (according to the hierarchy in Appendix 1). Otherwise, we will choose the next most important dichotomous outcome. Since we are interested in how authors present the results of their systematic reviews (for example, results obtained when combining the included studies), we will not collect information about absolute effects presented when describing individual studies included in the review, unless the comparison of interest includes only one trial.

### Search strategy

We will use the MEDLINE database to search for potentially eligible systematic reviews. We will use two distinct search strategies. First, we will use an adaptation of the systematic review filter, designed by the Health Information Research Unit of McMaster University to retrieve non-Cochrane systematic reviews. Second, we will use the Ovid ‘search by journal’ filter to identify Cochrane systematic reviews (Appendix 2). We will limit both searches to the year 2010. We will subsequently export citations to Endnote X4.0.2., and then into a web-based systematic review software (DistillerSR, Evidence Partners, Ottawa, Canada; https://systematic-review.ca) for eligibility screening and data extraction.

### Random sampling of citations

All identified citations will be stratified into Cochrane and non-Cochrane search results. We will obtain a random sample within each stratum and screen it according to our eligibility criteria. We will repeat the random sampling process as needed until reaching the final sample size, which will include the same number of Cochrane and non-Cochrane systematic reviews (see sample size section).

### Review process

We will undertake, in a duplicate and independent manner, title and abstract screening, full text screening and data abstraction. Irrespective of discrepancies, all studies selected at a title and abstract level will be included for the full text screening. Reviewers will resolve discrepancies at the level of full text and data abstraction by consensus, and if unsuccessful, with the help of a third reviewer. This arbitrator will independently review the article before discussing it with the reviewers. To ensure the validity and consistency of the process, we will conduct calibration exercises for each step of the process. We will also develop and pilot-test standardized forms and upload them onto the online systematic review software application. We will accompany all forms with detailed instructions. A core group will meet regularly to discuss progress and potential difficulties. We will create a study flow to describe the results of the different steps of the selection process.

### Data extraction

We will extract the following information from each included systematic review: study characteristics, quality of the systematic review, the calculation and reporting of absolute estimates of effects, and the interpretation of absolute estimates of effects.

#### Study characteristics

For all included systematic reviews, we will extract the following information:

1. Type of systematic review (Cochrane vs. not Cochrane);

2. Type of intervention (pharmacologic vs. other);

3. High-impact (*Journal of the Medical Association*, *New England Journal of Medicine*, *Lancet*, *Annals of Internal Medicine*, *Journal of the American Medical Association* and *PLoS Medicine*) vs. other journals;

4. Quality of the review;

5. Use of GRADE vs. not use of GRADE;

6. Statistical significance of the effect for the most patient-important outcome;

7. Source of funding (partially or completely funded by private for-profit organization or authors with financial conflicts of interest vs. others).

Specifically, we will collect information about whether the review was published in the five journals with the most journal citations (*Journal of the American Medical Association*, *New England Journal of Medicine*, *Annals of Internal Medicine*, *Lancet* and *PLoS Medicine*), the population and the intervention and control of interest. We will also extract information about source of funding (partially or completely funded by private for-profit organization vs. others) and the type of intervention (pharmacologic vs. other). We will note whether the systematic review used the GRADE approach; this includes whether authors provide a summary table, such as a summary of findings.

We will note whether the reviews include an absolute measure of effect (for example, ARR, NNT) for the most patient-important outcome for the selected comparison. We will also note this for any outcome other than the most patient-important, for both the comparison of interest and, if available, any other comparison. For the selected comparison, we will note whether the authors report benefits and harm outcomes and whether they report a measure of relative effect, a measure of absolute effect or both.

#### Quality of the systematic reviews

We will assess the methodological quality of eligible systematic reviews using the AMSTAR instrument [23].

#### Calculation and reporting of absolute estimate of effects

For those reviews that report at least one absolute effect estimate, we will record whether these estimates relate to the most patient-important outcome, any outcome within the comparison of interest or elsewhere in the full text. For the reviews that provide an absolute estimate for the most patient-important outcome, we will collect information about the type of measure (for example, risk difference, NNT) and the expression used when reporting if available (for example, risk reduced by 5%). We will explore how authors calculated the absolute estimates (for example, direct calculation from a meta-analysis); or modelled from baseline risk (for example, the median baseline risk from the included studies) and whether they state the calculation methods in their methods section. We will document the number of estimates of effect for different baseline risks and, if available, whether authors specify the source of these baseline estimates. If needed, we will contact authors for additional information.

#### Interpretation of absolute estimates of effects

Regarding interpretation, we will document whether authors discuss the fact that risk differences may vary to an important degree across subpopulations. We will also document the extent to which authors discuss this potential variability and their interpretation of the main effect of interest. Finally, we will assess whether the absolute estimates of effects are considered in the conclusion (a separate conclusion section or in the conclusion of a discussion section).

### Sample size

We will calculate the sample size on the basis of an examination of study characteristics associated with the reporting of absolute effects for the most patient-important outcome: we will undertake this by means of a regression analysis. In this model we will include seven study characteristics with a total of eight categories of variable. We will require ten events per category to examine the association. Previous estimates show that approximately 50% of systematic reviews report an absolute estimate of effect [16]. We will consider 40% as our best estimate when considering the most patient-important outcome. Therefore, we will probably require a sample size of approximately 200 systematic reviews for our study. To increase our confidence in our sample size estimate, we will conduct a pilot study of 60 systematic reviews to further inform the final sample size.

### Analysis

We will assess agreement between reviewers’ judgements of whether the investigators reported an absolute measure of effect for the most patient-important outcome. We will calculate chance-corrected agreement and interpret the results according to Landis and Koch guidelines (κ values of 0 to 0.20 represent slight agreement, 0.21 to 0.40 fair agreement, 0.41 to 0.60 moderate agreement, 0.61 to 0.80 substantial agreement, and greater than 0.80 almost perfect agreement) [24].

We will calculate the proportion of systematic reviews, reporting at least one absolute estimate of effect for the most patient-important outcome, for any outcome within the comparison of interest, or for any comparison and any dichotomous outcome excluding the comparison of interest. We will conduct two multivariable logistic regression analyses to examine the association between pre-specified study characteristics and, first, the reporting of an absolute estimate of effect for the most patient-important outcome and, second, the reporting of an absolute estimate of effect for any outcome within the comparison of interest.

We will also calculate the proportion of systematic reviews that report the method they used to calculate the absolute estimate. We will calculate the proportion of systematic reviews that discuss whether risk differences may vary across populations anywhere in the article. We will conduct two separate multivariable logistic regression analyses to examine the association with the pre-specified study characteristics and respectively these two features of risk difference calculation and interpretation.

In addition, we will calculate the proportion of systematic reviews that use, for the comparison of interest, relative measures for benefit outcomes and absolute measures for harm (‘mismatched framing’ henceforth). Treating the reporting of results with mismatched framing as the dependent variable, we will conduct multivariable logistic regression analyses to examine its association with the pre-specified study characteristics.

Our pre-specified study characteristics for the regression analyses are listed and ranked by importance. If there are sufficient events, we will include them all. Otherwise, we will include as many as possible according to our ten events-per-category rule (see section on sample size). The first two factors to be examined will be type of systematic review (Cochrane vs. not Cochrane) and use of GRADE. However if, as we will explain, there is excessive confounding, we will not include GRADE in the regression:

We hypothesize that systematic reviews are more likely to report absolute effects or report them appropriately if they: (i) are Cochrane reviews, (ii) use GRADE, (iii) are of better quality, (iv) achieve statistical significance for the most patient-important outcome, (v) do not receive funding from for-profit organizations or their authors have financial conflicts of interest, (vi) evaluate pharmacological interventions, (vii) are published in a high-impact journal (*Journal of American Medical Association*, *New England Journal of Medicine*, *The Lancet*, *British Medical Journal*, *Annals of Internal Medicine* and *Public Library of Science Medicine*).

Before the regressions, we will look at the proportion of Cochrane and not-Cochrane systematic reviews that include a GRADE approach. We suspect that GRADE might be reported seldom in non-Cochrane systematic reviews and relatively frequently in Cochrane systematic reviews. If this is the case, there will be excessive confounding between use of GRADE and Cochrane reviews and it will be inappropriate to include them in the same regression.

If this proves to be the case, we will use the following analytical approach. We will compare Cochrane systematic reviews with and without GRADE. If there is a significant difference, we will then compare Cochrane with GRADE vs. non-Cochrane and Cochrane without GRADE vs. non-Cochrane. The results of these analyses will determine whether we include Cochrane or GRADE or both in the complete regression.

## Discussion

### Main objectives of our study

Our review will establish the proportion of systematic reviews reporting absolute measures of effect and how Cochrane and non-Cochrane systematic reviews calculate, report and interpret these measures. We will evaluate the frequency in systematic reviews of mismatched framing, characterized by the use of relative measures for efficacy outcome and absolute measures for harm outcomes. Given the lack of information about this topic and the potential implications for decision-making in healthcare [18], we believe it is important to explore how this issue is managed in published systematic reviews. By publishing this protocol we are reflecting our commitment to making the objectives and design of methodological studies more transparent [19-22].

### Strengths and limitations

Our study has several strengths. First, we will use transparent and rigorous methods, including explicit eligibility criteria, sensitive search strategies and the use of standardized forms. We will pilot these forms and develop detailed instructions for both study screening and data extraction, and achieve near perfect agreement between reviewers before commencing study selection and data extraction. We will evaluate each of the reviews and extract data in duplicate and independently. Second, we will include both Cochrane and non-Cochrane systematic reviews and use, as opposed to previous studies, broad inclusion criteria to make our results more generalizable. Third, as in our previous projects [21], we will conduct a pilot study to improve the accuracy of our final sample size calculation. Fourth, we will explore issues that have not previously been addressed, including the type of absolute estimate reported and the method used for calculation. Finally, the feasibility of our study is increased due to the experience of our group in completing methodological studies involving large samples [25-27].

Our study has potential limitations. First, it will involve several reviewers’ judgements at each step of the process. The detailed instructions, piloting and calibration exercises described previously should help to minimize disagreement. Second, some of the reviewers are less experienced than others. To overcome this limitation, we will partner less experienced reviewers with those who are more experienced. We will also have a steering group that will meet regularly to discuss progress and potential difficulties.

### Previous research

Several studies have addressed the use of absolute effects in leading medical journals. Two of them explored this issue in individual studies observing that absolute estimates are very often not reported, especially in the abstract [14]. In the field of health inequalities research this percentage was strikingly low (9%) [15]. To our knowledge, only two studies have explored this issue in the context of systematic reviews. One study explored this issue in three of the top medical journals (*The Lancet*, *JAMA* and *BMJ*) showing that approximately 50% of the reviews included frequency data and one-third mismatched framing of benefit and harms [16]. This analysis was from a relatively limited sample of journals and the analysis did not explore the issue beyond the actual reporting of these estimates. Beller *et al.* have explored this issue but only in the abstract of systematic reviews [17].

While there is agreement that both patients and health professionals understand absolute estimates better than relative estimates, there is inconclusive evidence about the optimal way, in terms of understanding, for reporting absolute estimates. Some studies suggest that natural frequencies are preferable and others favour percentages [3,28,29]. Previous evaluations of absolute estimate reporting, regardless of the included designs, have not provided either detailed information about what type of absolute estimates are most often used in systematic reviews or what methods authors use to calculate these. To the extent that systematic reviews include the latter, their results are more likely to be well understood and, hence, optimally implemented.

### Implications

The findings of ARROW will inform the systematic review community about the current practice of absolute estimates reporting in both Cochrane and non-Cochrane reviews. Our findings may influence recommendations on reporting, conduct and interpretation of absolute estimates in this type of research design. Our results are likely to be of great interest for systematic review authors and developers, funding agencies, health decision makers, guideline developers, and journal editors.

## Appendix 1 Outcome importance hierarchy

I. Mortality;

a. All causes of mortality;

a. Disease-specific mortality.

II. Morbidity

a. Cardiovascular major morbid outcomes;

a. Other major morbid outcomes (for example, loss of vision, seizures, fracture, revascularization);

a. Recurrence, relapse, remission of cancer, disease-free survival;

a. Renal failure requiring dialysis;

a. Hospitalizations;

a. Infections;

a. Dermatological or rheumatologic disorders.

III. Symptoms, quality of life, or functional status (for example, failure to become pregnant, successful breastfeeding, depression);

IV. Surrogate outcomes (for example, diagnosis of tuberculosis, viral load, physical activity, weight loss, post-operative atrial fibrillation, cognitive function).

Categories I, II, or III but not category IV define a patient-important outcome. For a composite endpoint to be patient-important all its components have to be patient-important.

## Appendix 2 Search strategy

### Ovid MEDLINE search strategy for no Cochrane systematic reviews

1. Meta-Analysis as Topic/

2. meta analy$.tw.

3. metaanaly$.tw.

4. Meta-Analysis/

5. (systematic adj (review$1 or overview$1)).tw.

6. exp "Review Literature as Topic"/

7. 3 or 4 or 5 or 6

8. cochrane.ab.

9. embase.ab.

10.  (psychlit or psyclit).ab.

11.  (psychinfo or psycinfo).ab.

12.  (cinahl or cinhal).ab.

13.  science citation index.ab.

14.  bids.ab.

15.  cancerlit.ab.

16.  8 or 9 or 10 or 11 or 12 or 13 or 14 or 15

17.  reference list$.ab.

18.  bibliograph$.ab.

19.  hand-search$.ab.

20.  relevant journals.ab.

21.  manual search$.ab.

22.  17 or 18 or 19 or 20 or 21

23.  selection criteria.ab.

24.  data extraction.ab.

25.  23 or 24

26.  Review/

27.  25 and 26

28.  Comment/

29.  Letter/

30.  Editorial/

31.  animal/

32.  human/

33.  31 not (31 and 32)

34.  or/28-30,33

35.  7 or 16 or 22 or 27

36.  35 not 34

37.  36 not "cochrane database of systematic reviews".jn.

38.  limit 37 to yr = "2010"

### Ovid MEDLINE search strategy for Cochrane systematic reviews

1. "cochrane database of systematic reviews".jn.

2. limit 1 to yr = "2010"

## Abbreviations

ARR: Absolute risk reduction; NNT: Number needed to treat; RCT: Randomized controlled trial; RRR: Relative risk reduction.

## Competing interests

The authors declare that they have no competing interests.

## Authors’ contributions

PAC and GHG conceived the study. PAC, GHG, ACL, RBP, IN, EA and HJS developed a first design proposal. All authors provided feedback about the design. PAC wrote the first draft of the manuscript. All authors read and approved the final manuscript.
