# Correction: A methodological survey of the analysis, reporting and interpretation of Absolute Risk ReductiOn in systematic revieWs (ARROW): a study protocol

**DOI:** 10.1186/2046-4053-3-76

**Published:** 2014-07-17

**Authors:** Pablo Alonso-Coello, Alonso Carrasco-Labra, Romina Brignardello-Petersen, Ignacio Neumann, Elie A Akl, Xin Sun, Bradley C Johnston, Matthias Briel, Jason W Busse, Demián Glujovsky, Carlos E Granados, Alfonso Iorio, Affan Irfan, Laura Martínez García, Reem A Mustafa, Anggie Ramirez-Morera, Iván Solà, Kari A O Tikkinen, Shanil Ebrahim, Per O Vandvik, Yuqing Zhang, Anna Selva, Andrea J Sanabria, Oscar E Zazueta, Robin W M Vernooij, Holger J Schünemann, Gordon H Guyatt

**Affiliations:** 1 Iberoamerican Cochrane Centre, Institute of Biomedical Research (IIB Sant Pau), Barcelona, Spain; 2 Department of Clinical Epidemiology & Biostatistics, McMaster University, Hamilton, Canada; 3 Evidence-Based Dentistry Unit, Faculty of Dentistry, Universidad de Chile, Santiago, Chile; 4 Department of Medicine, Pontificia Universidad Católica de Chile, Santiago, Chile; 5 Department of Internal Medicine, American University of Beirut, Beirut, Lebanon; 6 Department of Medicine, State University of New York, Buffalo, NY, USA; 7 Chinese Evidence-based Medicine Centre, West China Hospital, Sichuan University, Chengdu, China; 8 Department of Anaesthesia and Pain Medicine, The Hospital for Sick Children, Institute of Health Policy, Management and Evaluation, University of Toronto, Toronto, Ontario, Canada; 9 Child Health Evaluative Sciences, The Hospital for Sick Children Research Institute, Toronto, Ontario, Canada; 10 Basel Institute for Clinical Epidemiology and Biostatistics, University Hospital Basel, Basel, Switzerland; 11 Department of Anaesthesia, McMaster University, Hamilton, Canada; 12 Argentine Cochrane Centre IECS (Institute for Clinical Effectiveness and Health Policy), Buenos Aires, Argentina; 13 Área de investigaciones, Facultad de Medicina, Universidad de La Sabana, Chía, Colombia; 14 Internal Medicine Residency Program, University of Illinois, Urbana-Champaign, Illinois, USA; 15 Department of Medicine and Nephrology, University Of Missouri-Kansas City, Missouri-Kansas City, Missouri, USA; 16 CCSS Permanent Medical Advisor, Health Care Development Division, IHCAI Foundation & Central America Cochrane, Costa Rica, Costa Rica; 17 Department of Urology, Helsinki University Central Hospital and University of Helsinki, Helsinki, Finland; 18 Institute of Health Policy, Management & Evaluation, University of Toronto, Toronto, Canada; 19 Norwegian Knowledge Centre for the Health Services, Oslo, Norway

## Correction

Following publication of our article [1], it has come to our attention that Laura Martínez García’s surname was displayed incorrectly. We publish this correction to update the author list, which is as follows:

Pablo Alonso-Coello, Alonso Carrasco-Labra, Romina Brignardello-Petersen, Ignacio Neumann, Elie A Akl, Xin Sun, Bradley C Johnston, Matthias Briel, Jason W Busse, Demián Glujovsky, Carlos E Granados, Alfonso Iorio, Affan Irfan, Laura Martínez García, Reem A Mustafa, Anggie Ramirez-Morera, Iván Solà, Kari A O Tikkinen, Shanil Ebrahim, Per O Vandvik, Yuqing Zhang, Anna Selva, Andrea J Sanabria, Oscar E Zazueta, Robin W M Vernooij, Holger J Schünemann and Gordon H Guyatt.
